# Enhanced Recovery Care versus Traditional Care after Surgery in Pediatric Patients with Inflammatory Bowel Disease: A Retrospective Case-Control Study

**DOI:** 10.3390/biomedicines10092209

**Published:** 2022-09-07

**Authors:** Valeria Dipasquale, Francesca Laganà, Serena Arrigo, Giuseppe Trimarchi, Carmelo Romeo, Giuseppe Navarra, Girolamo Mattioli, Paolo Gandullia, Claudio Romano

**Affiliations:** 1 Pediatric Gastroenterology and Cystic Fibrosis Unit, Department of Human Pathology in Adulthood and Childhood “G. Barresi”, University of Messina, 98122 Messina, Italy; 2 Pediatric Gastroenterology and Endoscopy Unit, Integrated Department of Pediatric and Hemato-Oncological Sciences, IRCCS “G. Gaslini” Children’s Hospital, 16147 Genoa, Italy; 3 Department of Economy, University of Messina, 98122 Messina, Italy; 4 Pediatric Surgery Unit, Department of Human Pathology in Adulthood and Childhood “G. Barresi”, University of Messina, 98122 Messina, Italy; 5 Department of Human Pathology in Adulthood and Childhood “G. Barresi”, Surgical Oncology Division, University of Messina, 98122 Messina, Italy; 6 Pediatric Surgery Unit, Department of Neuroscience, Rehabilitation, Ophthalmology, Genetics, Maternal and Child Health (DINOGMI), “G. Gaslini” Children’s Hospital, 16147 Genoa, Italy

**Keywords:** children, enhanced recovery after surgery, inflammatory bowel disease, length of hospital stay, postoperative complication, surgery

## Abstract

This study reports the outcomes of an enhanced recovery after surgery (ERAS) protocol in pediatric inflammatory bowel disease (IBD) surgery. Children who underwent surgery for IBD at two academic referral centers from January 2016 to June 2021 were included. Preoperative counseling, early enteral feeding (Impact^®^, Nestlé Health Science, and early mobilization were all part of the ERAS protocol. The outcomes (timing of first defecation, postoperative complications, and length of hospital stay (LOS)) were compared to traditional perioperative regimens (non-ERAS group). Thirty-three children who had 61 abdominal surgeries for IBD were included. Forty (65.5%) surgical procedures were included in the non-ERAS group, and 21 (34.5%) were included in the ERAS group. The postoperative complication rate was significantly lower in the ERAS group than in the non-ERAS group (29.6% vs. 55%, *p* = 0.049). The first defecation occurred earlier in the ERAS group than in the non-ERAS group (*p* < 0.001). There was no significant intergroup difference in the LOS. The implementation of ERAS in pediatric IBD surgery resulted in better outcomes than traditional perioperative care, especially in terms of postoperative complication rate and bowel function recovery. Further pediatric studies are needed to validate these findings and support ERAS application in children.

## 1. Introduction

Inflammatory bowel diseases (IBD; Crohn’s disease, CD; ulcerative colitis, UC; and IBD unclassified; IBDU) are chronic gastrointestinal diseases that can manifest in childhood in up to 30% of cases. In comparison to adults, children with IBD frequently have a more severe phenotype associated with the increased use of immunomodulatory therapy and the need for surgical treatment [1,2]. Indeed, surgery is required in around 20–30% of children within 10 years after diagnosis, and it results in a relevant improvement in quality of life [3,4,5]. Surgery is needed in children with UC who have acute severe colitis that is not responding to medical therapy and in those who have steroid-dependent disease, poor response to medical treatment, or growth and/or puberty delays. CD children require surgical treatment for complications such as strictures and fistulas. Improving perioperative care for pediatric patients is critical, because children’s physical stress reactions to traditional perioperative treatment are typically more severe than adults [6]. To reduce perioperative stress and organ dysfunction in surgical patients, Danish surgeon Henrik Kehlet originally proposed the notion of enhanced recovery after surgery (ERAS) in the 1990s [7]. Perioperative management traditionally included prolonged fasting and bed rest, bowel preparation, drainage tube and catheter insertion, and opioid-based analgesia. The evidence-based, patient-centered, multidisciplinary ERAS program is based on preoperative counseling, optimal anesthesia, a minimally invasive surgical approach, a shortened fasting period, early mobilization, and the nonroutine use of surgical drains [7,8]. ERAS was first used in adult colorectal surgery and then spread to other surgical specialties [7,9]. Studies in adults have shown that the use of ERAS protocols is associated with a shorter hospital stay, a lower incidence of postoperative complications, and faster convalescence [10,11]. The implementation of ERAS in pediatric surgical settings has only recently started [9]. Moreover, few studies have been conducted on ERAS in IBD patients, most likely due to concerns about placing these complicated patients on a more “aggressive” fast-track pathway [12]. The present study aims to determine the benefit of an ERAS protocol in a cohort of pediatric patients undergoing abdominal surgery for IBD and to compare the main outcomes with those of a traditional non-ERAS group of patients (who underwent IBD surgery prior to the implementation of the ERAS program in both institutions). 

## 2. Materials and Methods

### 2.1. ERAS Program

The ERAS protocol for IBD-related surgery included the following elements (Table 1 and Table 2): preoperative education and counseling; antibiotic prophylaxis; minimally invasive surgical approach whenever possible; multimodal analgesia; postoperative nausea and vomiting prophylaxis; early enteral feeding; less daily intravenous infusion volume; early removal of wound drainage and urinary catheters; and early mobilization. 

Preoperative education and counseling included the aims and procedures of the ERAS protocol, pain treatment strategies, parental expectations of this surgery, discharge criteria, and a follow-up plan. A nutritional evaluation was performed on all patients before surgery, and nutritional support was started on an individual basis before and/or after surgery. The preoperative fasting time was shortened by the cessation of clear fluids for at least 2 h as well as solid foods for 6 h before anesthesia. Antibiotic prophylaxis has been identified as one of the protective factors against surgical site infections [10,11]. Postoperative practice was early enteral feeding with a formula containing immune-modulating components such as L-arginine, omega-3 fatty acids, and nucleotides (Impact^®^, Nestlé Health Science, Switzerland) on the evening of surgery and a regular diet the next day, and early mobilization (including on-bed movements) the day after surgery was performed. Children had a low risk of deep venous thrombosis, so antithrombotic prophylaxis was not necessary. The data collection methods and service evaluation were designed retrospectively.

### 2.2. Population

In this retrospective, case-control study, the hospital medical records of pediatric patients (aged 0–18 years) who underwent abdominal surgery for IBD at two academic referral centers from January 2016 to June 2021 were reviewed. IBD-related surgery included the following procedures: (i) subtotal colectomy with ileostomy; (ii) proctocolectomy with ileal pouch anal-anastomosis with ileostomy; (iii) ileostomy closure; (iv) terminal ileostomy; and (v) ileocecal resection with or without ileostomy. IBD was diagnosed based on clinical features, laboratory tests, and endoscopic and histological findings [13]. The study was approved by the ethics committees of the participating sites in Italy and was performed in accordance with the Declaration of Helsinki. Informed consent was obtained from participants and/or their parents.

### 2.3. Data Collection

Preoperative, intraoperative, and postoperative data were collected for each procedure. Preoperative data included age at surgery, age at disease onset, IBD type, body mass index (BMI), pharmacological treatment, and nutritional support. Postoperative data included timing of first defecation (or stool output in the case of patients with ileostomy), complications, and length of hospital stay (LOS).

### 2.4. Outcome Measures

The primary outcome was the rate of postoperative complications. Secondary outcomes included the timing of first defecation, LOS, and the impact of preoperative factors such as IBD type, BMI, and pharmacological treatment on the rate of postoperative complications. The differences in each outcome between the ERAS group and the non-ERAS group were investigated. In the subgroup of patients who were malnourished (BMI < −2 standard deviation, SD) before surgery, the rate of postoperative complications and their association with preoperative nutritional support were investigated.

### 2.5. Statistical Analysis

Quantitative variables underwent a verification of normality using the Shapiro–Wilk test; in the case of acceptance of the hypothesis H0, the results were reported as mean and SD. In the case of refusal of H0, results were reported as median and range. Categorical variables were reported as frequency and percentage. The significance between patients stratified by ERAS (0 = No and 1 = Yes) and quantitative variables was assessed by a parametric approach (Student’s t-test) and a non-parametric approach (Mann–Whitney’s U test) based on the results of the Shapiro–Wilk test. The association among categorical variables was tested using the chi-square test or Fisher’s exact test when applicable. All statistical analyses were performed using R version 4.1.1 (R Foundation for Statistical Computing, Vienna, Austria). A *p*-value ≤ 0.05 was considered statistically significant.

## 3. Results

Results were identified in 33 children who underwent a total of 61 major abdominal surgery procedures for IBD. Overall preoperative and intraoperative data are summarized in Table 3. 

Postoperative complications occurred in 28 (45.9%) cases. They were surgical (i.e., intestinal obstruction, anastomotic dehiscence) in 33 (54.1%) cases and medical (i.e., fever, vomiting, wound infection) in the remaining ones. Complications were more frequent in UC patients than in CD or IBDU patients, but the difference was not statistically significant (*p* = 0.561). Similarly, no association was found between preoperative BMI or pharmacological treatment and the development of postoperative complications (*p* = 0.955 and *p* = 0.488, respectively). In the majority of procedures (*n* = 38, 62.3%) patients had the first defecation within 24 h. In 13 (21.3%) procedures, it occurred between 24 and 72 h, and in 10 (16.4%), after 72 h. 

### 3.1. ERAS vs. non-ERAS

**Figure 1:**
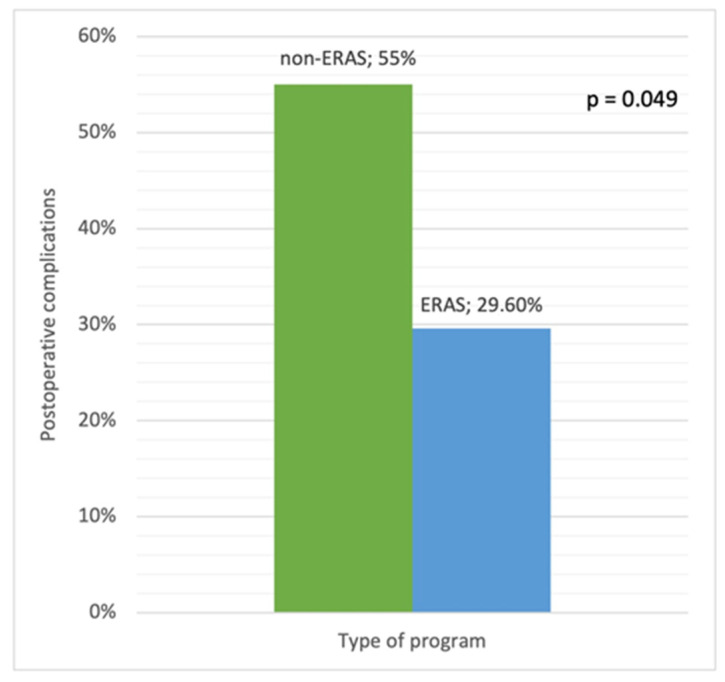
Postoperative complications in the ERAS and non-ERAS groups.

Forty (65.5%) surgical procedures were in the non-ERAS group or control group (i.e., patients who underwent IBD surgery prior to the implementation of the ERAS protocol in both institutions), and 21 (34.5%) were in the ERAS group. Postoperative complications were significantly less frequent in the ERAS group than in the control group (29.6% vs. 55%, *p* = 0.049) (Figure 1). 

The type of complication (medical vs. surgical) did not differ between the two groups (*p* = 0.557). The first defecation occurred earlier in the ERAS group than in the non-ERAS group (*p* < 0.001). In the ERAS group, all surgical procedures were followed by the first defecation ≤ 24 h after surgery, whereas in the control group, the first defecation occurred ≤ 24 h after surgery in 17 cases (42.5%), between 24 and 72 h in 13 cases (32.5%), and > 72 h in the remaining cases. However, this finding may have been biased, because the ERAS protocol was primarily applied to patients with protective ileostomy. Overall, the median LOS was 6 days ± 7.52, with no difference between the two groups (5 days ± 6.92 in the ERAS group and 8 days ± 7.80 in the non-ERAS group, *p* = 0.114).

### 3.2. Nutritional Intervention

In 14 (23%) cases, patients were undernourished (BMI < -2 SD) before surgery, and in five of them, postoperative complications (6/7 medical complications) occurred. In nine (64.3%) cases, a preoperative nutritional support was started, including enteral nutrition (*n* = 6), parenteral nutrition (*n* = 2), or both (*n* = 1). In this subgroup, there was no association between preoperative nutritional intervention and the rate of complications (*p* = 0.073).

## 4. Discussion

The present study showed that ERAS was successfully applied in two academic centers for children undergoing IBD-related abdominal surgery. In the currently available literature, there is a certain degree of heterogeneity in previous cohorts in terms of indications for surgery (cardiac, digestive, orthopedic, etc.) and types of surgical intervention, making direct comparison to the current results difficult. Moreover, the majority of previously published studies addressed the applicability of individual or different elements of ERAS rather than the application of a system. A pediatric-specific ERAS protocol for use in adolescents undergoing elective digestive surgical procedures has just recently been proposed [14]. The main finding of this study was that the ERAS group had lower rates of postoperative complications than the traditionally treated patients. Moreover, the recovery of bowel function (timing of first defecation) was faster in the ERAS group. This might be attributed to early enteral feeding and mobilization [15]. In particular, the perioperative use of an immunonutrition formula (Impact^®^, Nestlé Health Science, Switzerland), enriched with immunonutrients such as omega-3 fatty acids, arginine, and nucleotides, has previously shown clinical benefits, including a 36% to 91% reduction in the risk of postoperative infectious complications and a 2.6-day reduction in LOS [16,17]. In this study, the LOS in the ERAS group was approximately 3 days shorter than in the non-ERAS group, although this difference was not statistically significant. Previous findings in the pediatric IBD literature have been conflicting [18,19,20,21]. In a study of 51 pediatric IBD patients examined retrospectively at a single institution, ERAS patients (*n* = 28) had a nonsignificant reduction in mean LOS when compared to controls [18]. Postoperative early feeding was not part of the ERAS protocol. Neither the rate of complications nor the timing of first defecation after surgery were evaluated [18]. Similarly, a more recent retrospective review of a prospective database including 80 major abdominal procedures for IBD in 41 children found similar LOS in patients who used an ERAS protocol vs. those who did not [19]. However, when ERAS was implemented, both emergency department visits and readmissions within 30 days following surgery were significantly reduced [18]. In contrast, in a single-center retrospective study of 71 pediatric patients undergoing isolated laparoscopic-assisted ileocecectomy for CD, the use of a fast-track protocol in 45 of them resulted in decreased LOS and timing of first stool, with no significant difference in postoperative complications observed between the two groups [20]. In another cohort of 99 pediatric patients, predominantly IBD patients, who had elective gastrointestinal surgery (especially partial or total colectomy and ileocecectomy), LOS dropped from a median of 4 days pre-ERAS protocol (*n* = 52) to 3 days post-ERAS protocol (*n* = 56) without affecting complication rates or readmissions [21]. No difference in the complication rate was observed between the two groups [21].

The present study has some limitations. First, the design was retrospective, so the included cases and clinical management approaches were the confounding variables. Furthermore, the retrospective study design may have limited the clinical findings and management. Second, it had a limited sample size, making it difficult to draw conclusive conclusions. One strength of this study is that it included two academic centers, as opposed to most previous studies, which were single-center studies. Another strength of this study is that it focuses solely on IBD, allowing for comparisons of relatively similar cohorts before and after implementation, as well as the implementation of a pediatric-specific ERAS protocol. Despite its limitations, this study adds to the growing body of evidence supporting the use of more comprehensive ERAS pathways for pediatric IBD patients undergoing surgery. 

## 5. Conclusions

The current findings suggest that ERAS may provide better outcomes for children having abdominal surgery for IBD than traditional perioperative care. Adult research provided a solid foundation for pathway development as well as some supporting data to urge the creation of pediatric-specific recommendations. Because pediatric colorectal surgeries, surgical methods and indications, and preoperative risks differ, further prospective studies and pediatric-specific data on ERAS outcomes are required.

## Figures and Tables

**Table 1:** The ERAS protocol versus the traditional non-ERAS protocol.

	ERAS Protocol	Non-ERAS Protocol
**Preoperative management**		
Counseling	Education and illustration	Informed consent
Nutrition evaluation	Proper assessment	No requirement
Fasting	Fasting 6 h, water 2 h	Fasting 12 h, water 4 h
Sedative preanesthetic	No	Yes, if required
**Intraoperative management**		
Antibiotic prophylaxis	Yes	No requirement
Surgical approach	Minimally invasive	No requirement
Abdominal drainage tubes	Only if necessary	Routinely used
Pain	Comprehensive analgesic regimen	NSAIDs
Maintenance of normothermia	Always	No requirement
**Postoperative management**		
Diet	Day 1	After recanalization
Removal of urinary catheter	Day 1	No requirement
Removal of wound drainage	Day 2	Day 2–3
Nausea and vomiting prophylaxis	5-HT receptor antagonist	No requirement
Mobilization	Day 1	No requirement

ERAS, enhanced recovery after surgery; NSAID, nonsteroidal anti-inflammatory drugs.

**Table 2:** Components of the ERAS protocol for IBD.

Preoperative	Intraoperative	Postoperative
Education	Maintenance of body temperature	Pain treatment
Evaluation of nutrition	Drainages only if necessary	Early feeding after anesthesia awareness
No prolonged fasting		Early removal of catheters
Preventive antibiotics		Early mobilization
		Nausea and vomiting prophylaxis
		Compliance and follow-up

ERAS, enhanced recovery after surgery; IBD, inflammatory bowel disease.

**Table 3:** Pre- and intraoperative data of IBD-related surgeries.

Variable	Total (*n* = 61)
Age at surgery, y, median (range)	13.6 (4.6–17.8)
Age at IBD onset, y, median (range)	10.2 (0.6–16.8)
BMI, *n* (%)	
<−2 SD	14 (23)
−1.9–+1.9 SD	46 (75.4)
>+2 SD	1 (1.6)
Indication, *n* (%)	
UC	28 (45.9)
CD	28 (45.9)
CD-like IBDU	3 (4.9)
UC-like IBDU	2 (3.3)
Drugs, *n* (%)	
None	27 (44.3)
Steroids	6 (9.8)
Biologics ^a^	18 (29.5)
Both	10 (16.4)
Nutritional support, *n* (%)	
No	47 (77)
Enteral nutrition ^b^	8 (13.1)
Parenteral nutrition	4 (6.6)
Both	2 (3.3)
Approach, *n* (%)	
Laparoscopy	57 (93.4)
Laparotomy	4 (6.6)
Type, *n* (%)	
Proctocolectomies with ileal pouch anal-anastomosis with ileostomy	19
Ileostomy closures	16
Subtotal colectomies with ileostomy	14
Terminal ileostomies	5
Ileocecal resections without ileostomy	5
Ileocecal resection with ileostomy	1

IBD, inflammatory bowel disease; UC, ulcerative colitis; CD, Crohn’s disease; IBDU, IBD unclassified; SD, standard deviation. ^a^ anti-tumor necrosis factor-α agents. ^b^ Impact^®^, Nestlé Health Science, Switzerland.

## Data Availability

The datasets generated during the current study are available from the corresponding author on reasonable request.
